# Exploiting Stretchable Metallic Springs as Compliant Electrodes for Cylindrical Dielectric Elastomer Actuators (DEAs)

**DOI:** 10.3390/mi8110339

**Published:** 2017-11-22

**Authors:** Chien-Hao Liu, Po-Wen Lin, Jui-An Chen, Yi-Tsung Lee, Yuan-Ming Chang

**Affiliations:** Department of Mechanical Engineering, National Taiwan University, Taipei 10617, Taiwan; r06522525@ntu.edu.tw (P.-W.L.); b01501065@ntu.edu.tw (J.-A.C.); b01502100@ntu.edu.tw (Y.-T.L.); b01502065@ntu.edu.tw (Y.-M.C.)

**Keywords:** cylindrical dielectric elastomer actuators (DEAs), axial stretches, compliant electrodes, VHB 4905, high voltages

## Abstract

In recent years, dielectric elastomer actuators (DEAs) have been widely used in soft robots and artificial bio-medical applications. Most DEAs are composed of a thin dielectric elastomer layer sandwiched between two compliant electrodes. DEAs vary in their design to provide bending, torsional, and stretch/contraction motions under the application of high external voltages. Most compliant electrodes are made of carbon powders or thin metallic films. In situations involving large deformations or improper fabrication, the electrodes are susceptible to breakage and increased resistivity. The worst cases result in a loss of conductivity and functional failure. In this study, we developed a method by which to exploit stretchable metallic springs as compliant electrodes for cylindrical DEAs. This design was inspired by the extensibility of mechanical springs. The main advantage of this approach is the fact that the metallic spring-like compliant electrodes remain conductive and do not increase the stiffness as the tube-like DEAs elongate in the axial direction. This can be attributed to a reduction in thickness in the radial direction. The proposed cylindrical structure is composed of highly-stretchable VHB 4905 film folded within a hollow tube and then sandwiched between copper springs (inside and outside) to allow for stretching and contraction in the axial direction under the application of high DC voltages. We fabricated a prototype and evaluated the mechanical and electromechanical properties of the device experimentally using a high-voltage source of 9.9 kV. This device demonstrated a non-linear increase in axial stretching with an increase in applied voltage, reaching a maximum extension of 0.63 mm (axial strain of 2.35%) at applied voltage of 9.9 kV. Further miniaturization and the incorporation of compressive springs are expected to allow the implementation of the proposed method in soft micro-robots and bio-mimetic applications.

## 1. Introduction

Recent developments in soft robots and artificial biomimetic structures have greatly increased interest in dielectric bio-compatible elastomers capable of producing large deformations. These devices have been widely used in actuators and sensors. Recent applications include tunable lenses [1], micro-pumps [2], energy harvesting [3], transducers [4], soft robots [5,6,7,8], and artificial muscles [9,10,11] Most dielectric elastomer actuators (DEAs) are composed of stretchable materials, such as polymers sandwiched between two compliant electrodes that alter their shape under an applied external electrical stimuli. Cylindrical DEAs comprise hollow cylindrical elastomer tubes sandwiched between compliant electrodes inside and outside. Under an applied external voltage (stimulus), the electrostatic force exerted in the radial directions by the compliant electrodes squeezes the elastomer, which causes expansion in the axial direction. Different cylindrical configurations, such as tubes [12,13,14,15], helixes [16], and rolls [17], have been investigated to allow for bending and torsional and stretching/contraction motions. Spring-roll structures that exploit compressive springs demonstrate a combination of bending and linear extension, which is applicable to walking robots [18,19]. In addition to the axial deformations of cylindrical DEAs, a folded structure [20] and a multi-layer stack [21] have been developed to provide linear motion.

**Figure 1:**
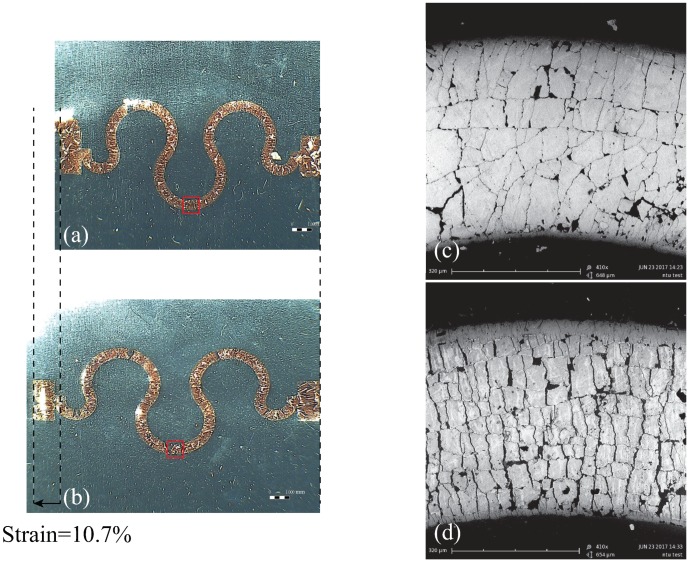
Photographs of unit cell of stretchable horseshoe-shape frequency-selective surfaces obtained using optical microscope with 12× magnification: (**a**) without stretching and (**b**) with stretching [34]; (**c**,**d**) scanning-electron-microscope (SEM) images of meandered parts at 410× magnification (while stretching, patterns broke apart along cracks, which resulted in loss of conductivity).

A variety of polymers and compositions have been studied to produce DEAs of higher performance. It has been demonstrated that compliant electrodes can affect the deformation of DEAs. Most compliant electrodes are made of carbon powder (graphene or carbon black) mixed with conductive glue [16,17,18,19,20,21,22,23,24] or metallic thin films applied to both sides of the elastomer [25,26,27]. In some situations, such as those involving large deformations, compliant electrodes are susceptible to breakage, increased stiffness, and increased resistance. In the worst cases, these devices lose all conductivity, which results in functional failure. Ideally, compliant electrodes should remain conductive and maintain good contact with elastomers during elongation. One shortcoming of metallic thin films is the fact that the Young’s modulus is far higher than that of the elastomer membrane, which can restrict DEA actuation. Take as an example the stretchable frequency-selective surfaces shown in Figure 1. The stretchable silicone substrate was coated with metallic horseshoe-shape patterns. Under an applied external force, it demonstrated longitudinal deformation resulting in strain of 10.7%, as shown in Figure 1a,b. When the device stretched, the metallic patterns broke and lost conductivity. A variety of methods have been investigated to create stretchable electrodes [13,14,15,16,17,18,19,20,21,22,23,24,25,26,27,28,29,30] and extend their length in order to cover the extrusions of the elastomers [31,32,33].

In this research, we adopted metallic springs as compliant electrodes for cylindrical DEAs to provide low resistance and stiffness under the effects of deformation. This design was inspired by the extensibility of mechanical springs. The proposed cylindrical DEA comprises a VHB 4905 (3M Company, Maplewood, MN, USA) thin film folded into a hollow tube and sandwiched between two individual copper springs inside and outside. VHBs are acrylic polymers widely used as dielectric elastomers with large expansion exceeding 100% strain [35,36] and high electrical breakdown strength [29]. Under external electrical stimuli, the proposed cylindrical DEAs provide axial deformation (stretches and contractions) similar to the linear-motion behavior of tube-like DEAs. The main advantage of the proposed spring-based compliant electrodes is the fact that they remain conductive and do not increase in stiffness throughout their range of motion. Preliminary work on this topic was reported in [37].

This paper is organized as follows. In the next section, we outline the configuration and theoretical model of the proposed cylindrical DEA and then describe the fabrication process. We then examine the mechanical properties of the fabricated device as well as electromechanical characteristics using a variable high-voltage source. Important results are summarized at the end of the paper.

## 2. Design and Simulations

### 2.1. Geometric Configuration

**Figure 2:**
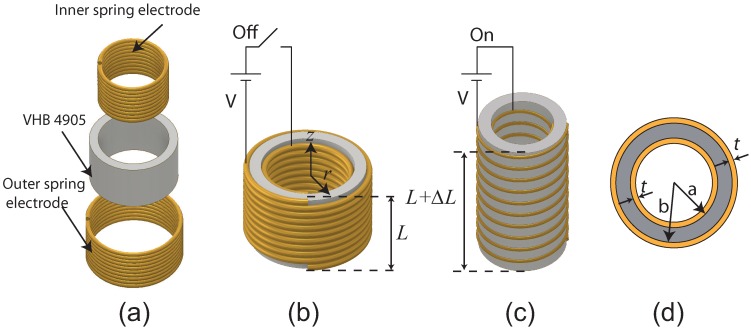
(**a**) Exploded view of proposed cylindrical Dielectric Elastomer Actuators (DEA) comprising VHB 4905 (3M Company, Maplewood, MN, USA) thin film folded into a hollow tube and sandwiched between two copper springs (inside and outside), which act as compliant electrodes; (**b**) without external electrical stimuli, device maintains its original shape; (**c**) under external electrical stimuli, device extends in the axial direction and decreases in the radial direction; (**d**) top view, where *a* is inner radius of VHB tube, *b* is outer radius of VHB tube, and *t* is wire diameter of springs (*L* and Δ*L* indicate original length before deformation and extended length of VHB tube).

Figure 2a presents an exploded view of the proposed cylindrical DEA comprising a thin VHB 4905-based tube (acting as an elastomer) sandwiched between copper springs inside and outside (acting as compliant electrodes). The inner copper spring was produced using a winder machine and then covered with 3M VHB 4905 thin film to a thickness of 0.5 mm and folded in the form of a hollow tube. The outer spring was then wrapped around the VHB tube using a winder machine. The geometrical dimensions of the DEA are presented in Table 1.

As mentioned in the previous section, we used copper springs as stretchable electrodes for their ability to remain conductive and retain low stiffness even as the elastomer is stretched. Without external electrical stimuli, the proposed cylindrical DEA retains its original shape, i.e., without deformation, as shown in Figure 2b. Figure 2c illustrates the situation involving the application of external electrical stimuli. The application of external DC voltage to the inner and outer copper springs creates an intense electrical field, penetrating the dielectric elastomer. The resulting electrostatic force squeezes the elastomer in the radial direction, causing it to expand in the axial direction. The axial deformation is proportional to the square of the applied DC voltage. Figure 2d presents a top-view image of the device.

### 2.2. Modeling

**Figure 3:**
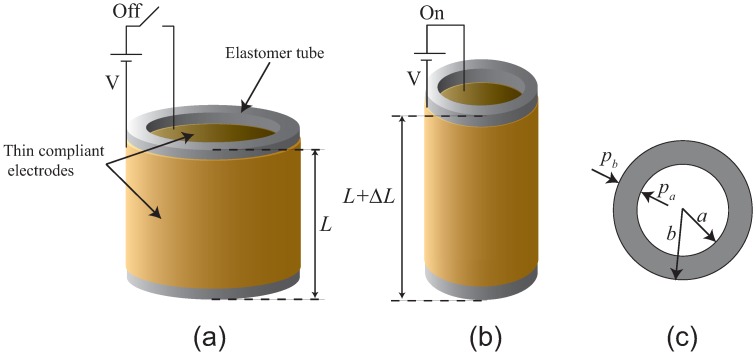
(**a**) Tube actuator with thin compliant electrodes on both sides of tube at its original length, i.e., without external electrical excitation; (**b**) under external excitation, actuator extends in the axial direction as indicated by ΔL; (**c**) top view of tube DEA where $p_{a}$ and $p_{b}$ refer to inner and outer pressures applied by compliant electrodes.

Under microscopic stretching and contraction, the distance between adjacent turns of the spring is relatively small when the cylindrical DEA is extended in the axial direction. The application of external electrical stimuli can result in an electric field uniformly distributed across the entire surface of the device. This is similar to the behavior of the tube actuator with thin compliant electrodes as shown in Figure 3. Thus, we assumed that the proposed spring-based cylindrical actuator could be modeled using the theoretical model of the tube actuator. As shown in Figure 3a, without application of external electrical excitation, the tube actuator maintains its original length, *L*. Under application of external voltage, V, the tube actuator deforms, such that the length is increased to ΔL. Based on the electromechanical model of the tube actuator in [12], the inner pressure, $p_{a}$, applied to the inner surface of the tube by the compliant electrodes can be expressed as follows:

(1) $$p_{a}=\frac{\epsilon V^{2}}{2ln^{2}\left(\frac{b}{a}\right)a^{2}b\left(b^{2}-a^{2}\right)}\times \sqrt{a^{6}+b^{6}-a^{2}b^{4}-b^{2}a^{4}+8ln\left(\frac{b}{a}\right)\left(b^{2}-a^{2}\right)a^{2}b^{2}+4ln^{2}\left(\frac{b}{a}\right)\left(b^{2}+a^{2}\right)a^{2}b^{2}}$$

where ϵ represents relative permittivity and V represents applied voltage. The outer pressure, $p_{b}$, applied to the two surfaces of the tube by the compliant electrodes is expressed as

(2) $$p_{b}=\frac{\epsilon V^{2}}{2ln^{2}\left(\frac{b}{a}\right)ab^{2}\left(b^{2}-a^{2}\right)}\times \sqrt{a^{6}+b^{6}-a^{2}b^{4}-b^{2}a^{4}+8ln\left(\frac{b}{a}\right)\left(b^{2}-a^{2}\right)a^{2}b^{2}+4ln^{2}\left(\frac{b}{a}\right)\left(b^{2}+a^{2}\right)a^{2}b^{2}}$$

and the extended length, ΔL, is expressed as

(3) $$\Delta L=L\frac{p_{b}b^{2}-p_{a}a^{2}}{Y(b^{2}-a^{2})}$$

where *Y* is the Young’s modulus. The analytical simulations of the proposed spring-based DEA based on the electromechanical model of the tube DEA will be presented in the later section. Note that this model is not necessarily applicable to large deformations.

## 3. Fabrication

**Figure 4:**
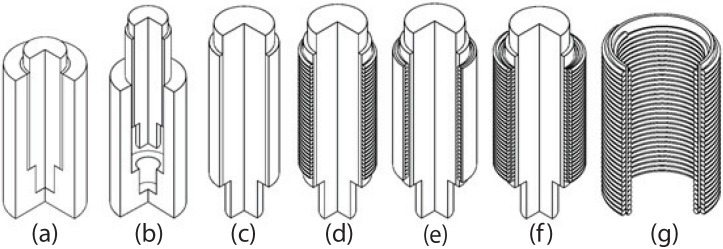
Fabrication process of proposed dual-spring cylindrical DEA: (**a**) cylindrical mold is first filled with Gypsum; (**b**,**c**) Gypsum axle is then demolded and stripped out from barrel; (**d**) first layer of compliant electrode is created by coiling copper wire around axle; (**e**) thin VHB 4905 film folded to cover cylindrical copper springs; (**f**) second layer of compliant electrode is created by coiling copper wire around outer surface of VHB film; (**g**) actuator comprising VHB film sandwiched by copper springs inside and outside is released from axle.

Figure 4a–g show the process used in the fabrication of the proposed cylindrical DEA. As shown in Figure 4a, a mold that includes an axle and a barrel is first created by 3D printing or machining.

**Figure 5:**
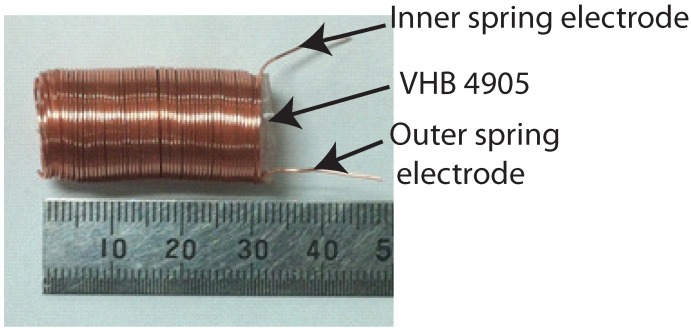
Photograph of fabricated spring-based-electrode cylindrical DEA.

The inside of the barrel was filled with Gypsum after being smeared with Vaseline for later mode-stripping. The proportion of Gypsum to water was optimized to enable a short curing time while ensuring strength sufficient for the fabrication of the actuator. After curing, the axle was demolded with Gypsum from the barrel, as shown in Figure 4c. The first layer of the compliant electrode was created by coiling copper wire over the Gypsum in a spring shape to form a uniform electrode, as shown in Figure 4d. A thin VHB 4905 film was then carefully folded to cover the cylindrical copper strings, as shown in Figure 4e. The second layer of the compliant electrode was then produced by coiling the copper wire over the outer surface of the VHB film, as shown in Figure 4f. Finally, the actuator was released from the axle by demolding the Gypsum with hot water, as shown in Figure 4g. Figure 5 presents a photograph of the fabricated cylindrical DEA with a total length of 30 mm.

## 4. Characterization of Mechanical Properties

**Figure 6:**
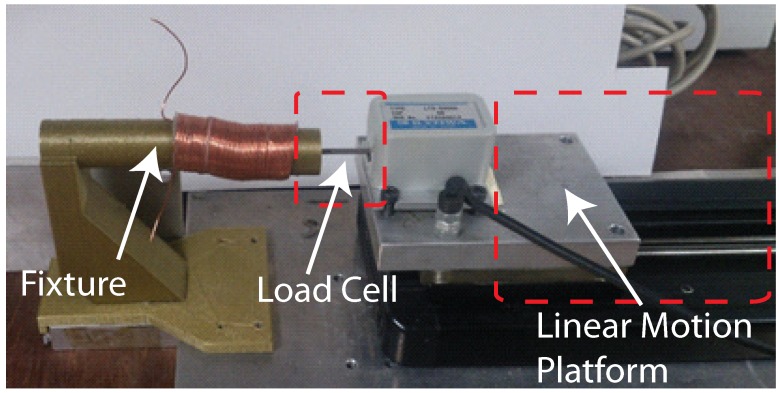
Experimental testbed for characterizing mechanical properties of proposed cylindrical DEA (stress-strain relation obtained by measuring axial deformations under external axial load).

Figure 6 presents the experimental testbed used to characterize the mechanical properties of the cylindrical DEA. The device being tested was placed with one end fixed and the other end in contact with a load cell mounted on a moving platform. The force applied to the DEA and the deformations in the DEA induced by moving the platform could be measured using the load cell with the distance moved by the platform.

**Figure 7:**
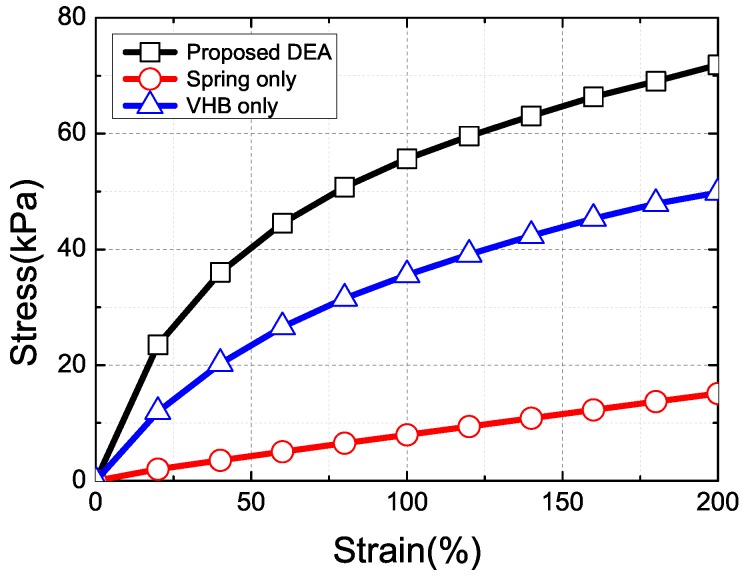
Measured stress-strain characteristics of entire device, individual copper springs, and VHB film, respectively.

We obtained stress-strain relation of the fabricated cylindrical DEA by measuring the axial strain under axial loads, as shown in Figure 7. The stress was defined as the force per unit area and the strain was defined as the ratio of the extended length to the original length. The elastic coefficient of the cylindrical DEA is a combination of the elastic coefficients of the dielectric elastomer and the compliant electrodes; therefore, we examined the individual stress-strain relationships of the VHB film and copper spring separately, as showed in Figure 7.

The proposed cylindrical DEA demonstrated a non-linear stress-strain relation similar to that of the VHB film. The individual copper spring presented a linear stress-strain relation with little overall impact on the actuator. In other words, the non-linear behavior of the proposed DEA was due mostly to the nonlinear properties of VHB film. The Young’s modulus of the proposed metallic springs was lower than that of the electrodes coated with a metallic film, falling within the GPa range suitable for actuation applications.

## 5. Electromechanical Properties

### 5.1. Experiment Setup

**Figure 8:**
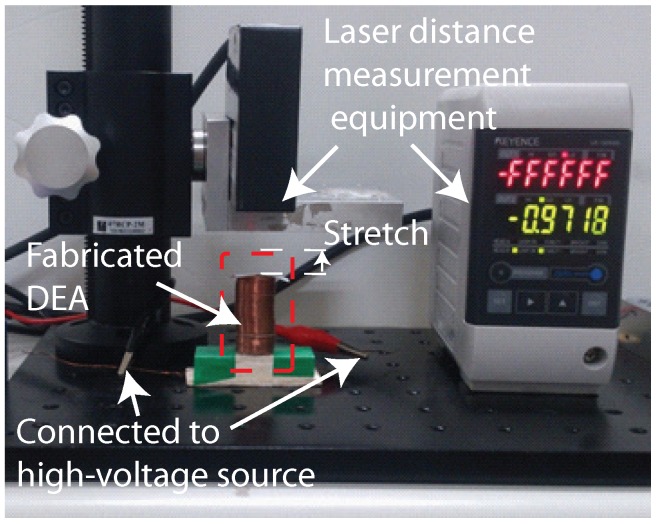
Experiment setup used to characterize electromechanical properties of dielectric DEA: device placed vertically and connected to variable voltage source of 9.9 kV; under application of external voltage, device extended vertically and axial deformations measured using laser-based distance measurement device.

Figure 8 presents the setup used to examine the electromechanical properties of the fabricated cylindrical DEAs. The device was placed vertically and connected to a variable voltage source of 9.9 kV. Laser-based distance measuring equipment (KEYENCE LK-Navigator, Keyence Corporation, Osaka, Japan) with accuracy of 0.1 μm was used to detect vertical deformations in cylindrical DEA at the micro-scale. All of the experiments were conducted on an optical table to reduce the influence of vibrations. A high voltage power supply, EMCO USBHV (XP power, Sunnyvale, CA, USA), was used to stimulate the device, as shown in Figure 8. The red conducting wire was connected the power supply via the inner copper spring whereas the black conducting wire was connected the power supply via the outside spring. Manually switching the power supply on and off generated periodic excitation with an amplitude of 9.9 kV and duty cycle of 25% (total period of 240 s). In each cycle, high DC voltage was applied to the cylindrical DEA in the first 60 s and removed in the last 180 s.

### 5.2. Experiment Results

**Figure 9:**
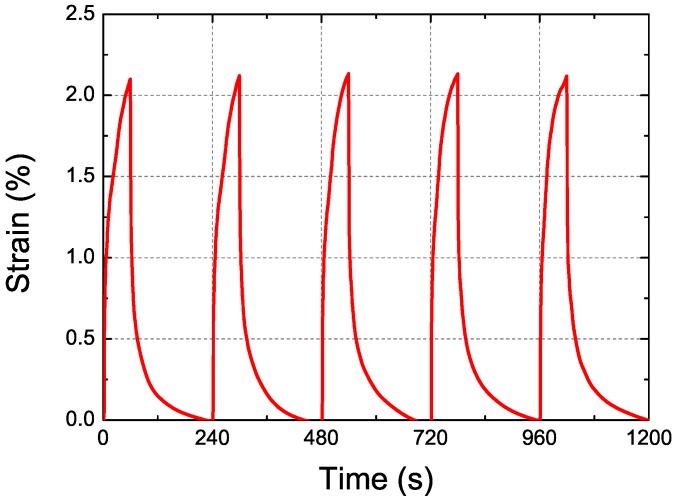
Measured axial strain in cylindrical DEA as function of time: periodic electrical stimuli with amplitude of 9.9 kV and duty cycle of 25%. Each cycle of time-domain response included charging process of 60 s and discharging process of 180 s. Cylindrical DEA extended during charging process and contracted during discharging process.

Figure 9 presents the measured axial strain in the proposed cylindrical DEA in the time domain. Under high voltage excitation during the first 60 s of each cycle, the cylindrical DEA extended to its maximum. When the applied voltage was removed in the last 180 s of each cycle, the cylindrical DEA returned to its original shape. The same behaviors were observed in other cycles; i.e., the cylindrical DEA extended when external voltage was applied and contracted when the external voltage was removed. The strain-time response was a periodic function indicating that the proposed cylindrical DEA provides consistent extensions and contractions.

### 5.3. Effects of Varying Applied Voltage

**Figure 10:**
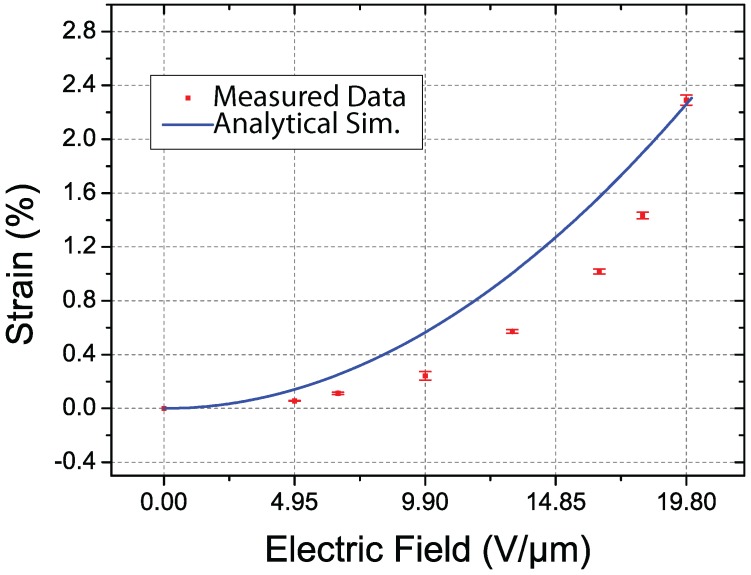
Simulated and measured axial strain values in proposed cylindrical DEA under external electrical stimuli ranging from 0 V to 9.9 kV, which corresponds to an electric field ranging from 0 to 19.8 V/μm (curve indicates simulation results obtained using the theoretical model described in Section 2.2; red points indicate average measured strain values and error bars indicate one standard deviation).

Based on the aforementioned theoretical model, we determined that the axial deformations in the cylindrical DEA were proportional to the square of the applied external voltage. We repeated the experiments in Section 5.2 using voltages ranging from 0 V to 9.9 kV, which corresponds to an electric field ranging from 0 to 19.8 V/μm. Figure 10 presents the simulated and measured axial strain values in the proposed cylindrical DEA. The curve represents the results obtained from the simulations in Section 2.2. The red points indicate the average measured strain values and the error bars indicate one standard deviation, where the parameters exploited in the simulation are reported in Table 2. Clearly, the measurement results are in line with those obtained in simulations. Any discrepancy between the two can be attributed to the non-linear behavior of the fabricated device, fabrication errors, and the assumption of a uniform distribution of electric field intensities.

## 6. Discussion

In the previous sections, we demonstrate that the proposed cylindrical DEA undergoes deformation under external electrical stimuli, resulting in a maximum axial strain of 2.35% under an external electric field of 20 V/μm. The deformations would be expected to increase with the strength of voltage. Nonetheless, we limited the maximum output voltage to 9 kV in order to prevent breakdown due to excessive voltage. One alternative approach to increasing the amount of deformation would be to pre-strain the VHB film. We opted not to examine this avenue due to the difficulties involved in introducing strain using the fabrication processes described in Section 3.

Table 3 presents a comparison of axial deformation in various designs reported in previous studies. The DEA proposed in this study has a tube-like configuration and stretchable-spring compliant electrodes; therefore, the axial strain was several times larger than that of actuators in a tube [12,13] or roll [17] configuration under the same electric field excitation. This can be attributed to the lack of deformation in film-coated compliant electrodes and multi-layer compliant electrodes, which tend to restrict axial deformation. The primary advantage of the proposed device is the fact that the spring electrodes remain conductive even as the dielectric elastomer extends. Electrodes based on metallic thin films are susceptible to breakage and a loss of conductivity under large strain. Nonetheless, the proposed cylindrical DEA has two weaknesses: (1) a portion of the device is uncovered as the spring electrodes extend; and (2) spring rigidity could hinder actuation of the dielectric elastomer. Therefore, compared with the co-axial tube [14], folded [20] and helix [16] DEAs, the proposed device is capable of large axial deformations under low electric excitation. This can be attributed to the fact that the geometric configuration was developed for compliant electrodes and the extension of elastomers in the axial direction. These issues are under investigation.

Overall, the proposed cylindrical DEA demonstrates good electromechanical performance with linear motion at the micro-scale. The proposed device could be scaled down using precision microelectromechanical system (MEMS) fabrication techniques. Miniaturized cylindrical DEAs could be used as cylindrical legs or arms for soft micro-robots. The proposed approach could also be incorporated with compressive springs to provide additional bending motions for use as swing legs. In addition, small cylindrical DEAs could be used to achieve linear movements in bio-mimetic structures [6].

## 7. Conclusions

This paper describes the use of copper springs as compliant electrodes for cylindrical dielectric elastomer actuators. Our objective was to preserve the conductivity and low stiffness of the compliant electrode under deformation. We fabricated a prototype and evaluated the mechanical and electromechanical properties of the device experimentally using a high-voltage source of 9.9 kV. Our results demonstrate that the proposed cylindrical DEA stretches under external electric stimuli and contracts following removal of the stimuli. Further miniaturization and the incorporation of compressive springs is expected to allow the implementation of the proposed method in soft micro-robots and bio-mimetic applications.

## Figures and Tables

**Table 1:** Geometrical dimensions of proposed cylindrical DEA.

	Meaning	Value (mm)
*d*	Diameter of springs	0.5
*a*	Inner radius of VHB film	8
*b*	Outer radius of VHB film	8.5
*L*	Total length	30

**Table 2:** Parameters exploited in the simulations.

Parameters	Value
ϵ	6 [38]
*a*	8 mm
*b*	8.5 mm
*L*	30 mm
*Y*	18 kPa [39]

**Table 3:** Comparison of axial deformations in various existing DEA structures.

	Axial Strain (%)	E-Field (V/μm)	Max. Axial Strain (%)	E-Field (V/μm)
Cylindrical coextruded tube [13]	0.7	20	1.9	36
Cylindrical tube [12]	0.3	20	4.5	100
co-axial tube [14]	4	20	7	24
Helix [16]		20	5	14
Roll [17]	1.8	20	12.77	58
Folded [20]		20	15.5	12
Our work	2.35	20	2.35	20
